# Ultrasonographic predictors of extubation success in children: diaphragmatic, lung, and laryngeal assessment—a prospective pilot cohort study

**DOI:** 10.1007/s00431-025-06677-6

**Published:** 2025-12-27

**Authors:** Ahmed R. Rezk, Hanan M. Ibrahim, Mahmoud Yousry, Assad Gamal, Sondos M. Magdy

**Affiliations:** 1 Pediatric Intensive Care Unit, Children’s Hospital, Ain Shams University, Cairo, Egypt; 2 Radiology Department, Children’s Hospital, Ain Shams University, Cairo, Egypt

**Keywords:** Extubation failure, Ultrasonography, Pediatric, Critical care, POCUS, Pilot study

## Abstract

Extubation failure, occurring in 3–22% of pediatric intensive care unit (PICU) patients, contributes to increased morbidity, prolonged hospitalization, and healthcare costs. Point-of-care ultrasound (POCUS) provides a bedside, radiation-free method to assess extubation readiness. This pilot study evaluated the predictive value of lung, diaphragmatic, and laryngeal ultrasound for extubation success in mechanically ventilated children. In this prospective pilot study, 30 children (22 males, 8 females; aged 1 month–14 years) ventilated for > 24 h were enrolled. Patients with congenital diaphragmatic, pulmonary, or laryngeal abnormalities were excluded. Ultrasound examinations of the diaphragm, lungs, and larynx were performed within 24 h before and after extubation. Patients were classified as successful or failed extubation (reintubation within 48 h). Receiver operating characteristic (ROC) curve analysis was used to determine optimal cut-off values. Extubation succeeded in 18 (60%) and failed in 12 (40%) patients. Diaphragmatic excursion > 5.5 mm was the strongest predictor (sensitivity 100%, specificity 91.7%). Diaphragmatic thickening fraction > 29% (sensitivity 88.9%, specificity 66.7%); lung ultrasound score < 16.5 (sensitivity and specificity 80%); and body mass index > 14.8 (sensitivity 100%, specificity 63.6%) were also significant predictors. Reduced laryngeal air width difference moderately predicted failure (specificity 83.3%, *p* = 0.025). *Conclusion*: Diaphragmatic excursion and thickening fraction are the most accurate ultrasound predictors of extubation success, with lung and laryngeal assessments providing complementary insights. Combining these modalities may improve bedside evaluation of extubation readiness in critically ill children.

**Table:** 

**What is Known:** • *Extubation failure remains frequent in pediatric intensive care units, and currently used clinical predictors have limited reliability.* • *Bedside ultrasonography enables non-invasive assessment of diaphragmatic, pulmonary, and laryngeal function in critically ill children.*
**What is New:** • *This pilot study integrates diaphragmatic, lung, and laryngeal ultrasonography into a single bedside extubation assessment strategy in children.* • *Diaphragmatic excursion and diaphragmatic thickening fraction emerged as the strongest ultrasound predictors of extubation success.*

**What is Known:**

• *Extubation failure remains frequent in pediatric intensive care units, and currently used clinical predictors have limited reliability.*

• *Bedside ultrasonography enables non-invasive assessment of diaphragmatic, pulmonary, and laryngeal function in critically ill children.*

**What is New:**

• *This pilot study integrates diaphragmatic, lung, and laryngeal ultrasonography into a single bedside extubation assessment strategy in children.*

• *Diaphragmatic excursion and diaphragmatic thickening fraction emerged as the strongest ultrasound predictors of extubation success.*

## Introduction

Mechanical ventilation (MV) is an essential component of pediatric intensive care, yet prolonged dependence can lead to significant complications including ventilator-associated pneumonia, cardiovascular compromise, and airway trauma. The duration of MV correlates directly with morbidity, mortality, and prolonged hospitalization [1].

Extubation failure—defined as the need for re-intubation within 48 h after planned extubation—remains a major clinical challenge, with reported rates between 3 and 22% in children irrespective of illness severity. Failed extubation increases PICU length of stay, costs, and mortality [2]. Despite various physiological indices, a universally reliable predictor of extubation readiness is lacking.

Point-of-care ultrasound (POCUS) has emerged as a valuable bedside tool that provides real-time, radiation-free assessment of respiratory function. Both pulmonary aeration and diaphragmatic contractility—two principal determinants of extubation success—can be accurately evaluated using ultrasound [3]. Laryngeal ultrasound, by measuring the laryngeal air-column width difference (LACWD), may also identify patients at risk of post-extubation airway compromise [4].

Previous pediatric studies have investigated these ultrasound parameters separately. To our knowledge, this pilot study is the first to integrate diaphragmatic, lung, and laryngeal ultrasound in a unified framework to predict extubation outcomes in children. This combined approach may enhance the clinical utility of POCUS for extubation readiness assessment in the PICU**.**

## Materials and methods

### Participants and eligibility

All consecutive PICU admissions were screened for eligibility. Children aged 1 month to 14 years who required invasive mechanical ventilation for more than 24 h via an endotracheal tube and were judged clinically ready for extubation by the treating team were eligible. Patients were excluded if they had congenital diaphragmatic, pulmonary, or laryngeal abnormalities; were primarily intubated for stridor (to avoid confounding laryngeal ultrasound interpretation, as pre-existing subglottic or glottic pathology could independently alter air-column measurements); or had pre-existing neuromuscular disease. Thirty patients (22 males, 8 females) fulfilled the criteria and were enrolled.

A sample size of 30 patients was estimated using PASS 15 software (power 80%, *α* = 0.05). Based on Alam et al. (2022) reporting an AUC = 0.83 for diaphragmatic ultrasound to predict extubation success with an anticipated success rate of 80%, this number was considered adequate for pilot validation.

### Clinical and laboratory assessment

For each patient, demographic characteristics (age, sex, body-mass index (BMI)), diagnosis, and duration of intubation were recorded, along with history of prior intubation, post-extubation respiratory support, and medication use (corticosteroids, diuretics). Physical examination emphasized respiratory findings, and post-extubation stridor was graded using the Westley Croup Score [5]. The appropriateness of endotracheal-tube size was confirmed using the Cole formula [6]. Arterial blood-gas parameters were collected before and after extubation. Nutritional status and fluid balance were assessed, with furosemide use serving as an indicator of possible fluid overload.

### Ultrasound examination

All ultrasound studies were performed on a Samsung Medison HM70A portable machine. In our institution, pediatric POCUS is primarily undertaken by radiologists because bedside ultrasound training for intensivists is still being integrated into local practice. Accordingly, a single consultant radiologist with extensive pediatric expertise performed all scans, assisted by a pediatric intensivist trained under direct supervision to ensure reproducibility and facilitate future clinical adoption. Each parameter was measured three times, and the mean of the three readings was used for analysis. Two ultrasound sessions were performed for every child—Peri-extubation imaging was scheduled within 24 h before extubation (during the spontaneous breathing trial window) and within 24 h after extubation. This interval was selected to balance feasibility with clinical relevance and to align with previous pediatric studies that used pre-extubation and ≤24-h post-extubation ultrasound for diaphragmatic and pulmonary assessment [8, 9]. The post-extubation scan was intended to capture early laryngeal edema and lung re-aeration changes associated with extubation outcomes. Future work should standardize a shorter pre-extubation interval (≤2–4 h) to optimize predictive validity [10].

Uniform imaging settings were maintained throughout all scans. Gain and depth were adjusted to optimize visualization without signal saturation, using 12–4 MHz linear probes for superficial structures (larynx, lung surface, diaphragm thickness) and 3–5 MHz curvilinear probes for diaphragmatic excursion. Examinations were conducted in the supine position with neck extension for laryngeal ultrasound and semi-recumbent (30° head elevation) for lung and diaphragmatic scans. Measurements were standardized at end-inspiration and end-expiration. Oropharyngeal suctioning was performed, and the ETT cuff was deflated before measurements to minimize artefacts.

Laryngeal ultrasound: A 12–4 MHz linear probe was placed transversely over the cricothyroid membrane with the patient’s neck slightly extended. The laryngeal air-column width was measured as the distance between the inner margins of the hyperechoic air–mucosa interface at end-inspiration.

The air-column width difference (ACWD) was calculated as the difference between the pre-extubation width (with the endotracheal tube in situ and cuff deflated) and the post-extubation width. Air around the tube = pre-extubation air-column diameter – ETT outer diameter, representing the air leak margin around the tube.

This approach was chosen to quantify peri-extubation changes in subglottic airway patency and to anticipate post-extubation stridor within 48 h.

This protocol represents a pediatric adaptation of methods described by Tsai WW et al. [11] and Haaksma Burton L et al. [9], differing from adult studies that measure the ACWD with the cuff alternately inflated and deflated before extubation. The rationale for this modification has been clarified to maintain physiological relevance while minimizing patient manipulation.

Laryngeal ultrasound was performed by placing the linear probe transversely across the cricothyroid membrane. The air-column diameter was measured at end-inspiration before and after extubation, and two derived indices were calculated:

Laryngeal air-column width difference (LACWD) = pre-extubation – post-extubation air-column diameter.

Lung ultrasound (LUS) followed a 12-zone protocol (anterior, lateral, posterior; upper and lower; bilaterally). Each zone was scored by its most severe finding:

0 = A-lines (normal), 1 = multiple discrete B-lines, 2 = coalescent B-lines, 3 = consolidation.

The global LUS aeration score was the sum of all regions (0–36 for 12-zone). At the time of data collection, this 12-zone scoring approach represented the standard practice in pediatric critical care. The recently published ESICM–ESPNIC 2025 international guidelines [7] recommend a 10-zone LUS aeration score for infants younger than 18 months, while retaining the 12-zone protocol for older children. Our study predated these recommendations.

Diaphragmatic ultrasound focused on the right hemidiaphragm. For the diaphragmatic thickening fraction (DTF), a linear probe was placed at the 8th–9th intercostal space between the anterior and mid-axillary lines; diaphragm thickness was measured from pleural to peritoneal line at end-expiration (Te) and end-inspiration (Ti), and DTF (%) = [(Ti − Te)/Te] × 100.

For diaphragmatic excursion (DE) and time-to-peak inspiratory amplitude (TPIA), a curvilinear probe in M-mode was positioned subcostally through the liver acoustic window, and vertical displacement (cm) and contraction-to-peak interval (s) were recorded.

## Outcome definitions and confounders

Extubation success was defined as the absence of re-intubation within 48 h, and failure as re-intubation within that period. All patients were free from sedatives and neuromuscular blockers for at least 6 h before assessment. Potential confounders such as corticosteroid administration, nutrition, and fluid status were documented for subsequent analysis.

## Statistical analysis

Data were analyzed using SPSS v27 (IBM Corp., Armonk, NY, USA). Continuous variables with normal distribution are reported as mean ± standard deviation (SD) and categorical variables as frequency (%). Normality was assessed with the Kolmogorov–Smirnov and Shapiro–Wilk tests. Between-group differences were analyzed using the independent-samples *t*-test or the chi-square test, as appropriate. Correlations between continuous variables were evaluated with the Spearman rank correlation coefficient. Receiver-operating-characteristic (ROC) analysis identified optimal cut-off values for key ultrasound predictors. Statistical significance was defined as a two-tailed *p* < 0.05 (95% confidence interval).

## Ethical considerations

The study was approved by the Faculty of Medicine, Ain Shams University Research Ethics Committee (FMASU REC No. FMASU MD103/2023; FWA No. FWA000017585). Written informed consent was obtained from the parents or legal guardians of all participants in accordance with the Declaration of Helsinki (2013 revision).

## Results

Thirty children were enrolled in this pilot study, including 22 males (73.3%) and 8 females (26.7%), with a mean age of 51.5 ± 58.6 months. The mean duration of intubation was 10.3 ± 9.9 days, and the average body mass index (BMI) was 15.8 ± 5.4 kg/m [2]. Extubation was successful in 18 patients (60%), while 12 patients (40%) required reintubation within 48 h and were classified as failures. Following extubation, 26 children (86.7%) required additional respiratory support—most commonly high-flow nasal cannula in 14 (46.7%), continuous positive airway pressure in 9 (30.0%), and low-flow oxygen in 3 (10.0%)—reflecting a stepwise weaning strategy in the unit as shown in Table 1.

**Table 1:** Demographic and clinical characteristics of the study cohort (*n* = 30)

Variable	Mean ± SD or *n* (%)
Age (months)	51.5 ± 58.6
Male sex	22 (73.3)
Weight (kg)	15.4 ± 16.0
Height (cm)	88.7 ± 35.6
BMI (kg/m^2^)	15.8 ± 5.4
Duration of intubation (days)	10.3 ± 9.9
Post-extubation support	HFNC 14 (46.7)
	CPAP 9 (30.0)
	low-flow 3 (10.0)
	none 4 (13.3)
Extubation success	18 (60.0)
Extubation failure	12 (40.0)

Blood cultures were negative in 24 cases (80%), with occasional isolation of *Escherichia coli*, *Acinetobacter*, *Pseudomonas*, *Klebsiella pneumoniae*, and *Candida* species. Sputum cultures were negative in 22 (73.3%) children, while the respiratory pathogen panel, performed in selected cases, detected rhinovirus in 4 (13.3%), non-COVID coronavirus in 2 (6.7%), and human metapneumovirus in 1 (3.3%). None of the microbiological findings differed significantly between extubation success and failure groups. Similarly, pre- and post-extubation arterial blood gases revealed no significant differences in pH, PaCO₂, or bicarbonate levels, indicating stable gas exchange across groups (all *p* > 0.05).

An exploratory comparison of ultrasound findings before and after extubation was conducted to observe short-term physiological changes in diaphragmatic function, lung aeration, and airway caliber, rather than to predict extubation outcome as shown in Table 2.

**Table 2:** Ultrasound parameters before and after extubation

Parameter	Before mean ± SD	After mean ± SD	*p*-value
Air-passing diameter (cm)	0.63 ± 0.18	0.49 ± 0.16	< 0.001 **
LUS aeration score	21.0 ± 6.0	15.0 ± 9.6	0.036 *
Diaphragmatic thickening fraction (%)	43.1 ± 20.3	45.1 ± 36.4	0.717
Diaphragmatic excursion (cm)	1.02 ± 0.84	0.83 ± 0.35	0.160
Time to peak inspiratory amplitude (s)	0.32 ± 0.13	0.32 ± 0.13	0.892

Using: Independent *T*-test for mean ± SD, *p*-value > 0.05 is insignificant; *p-value < 0.05 is significant; **p-value < 0.01 is highly significant

Ultrasound findings before and after extubation showed distinct respiratory pattern changes. Laryngeal ultrasound demonstrated a significant post-extubation decrease in air-passing diameter (0.63 ± 0.18 cm before vs. 0.49 ± 0.16 cm after extubation; *p* < 0.001), consistent with transient airway narrowing. The global lung ultrasound (LUS) aeration score improved significantly following extubation (21.0 ± 6.0 vs. 15.0 ± 9.6; *p* = 0.036), reflecting better lung aeration after tube removal. Diaphragmatic indices—thickening fraction (DTF), excursion (DE), and time-to-peak inspiratory amplitude (TPIA)—did not differ significantly pre- and post-extubation (*p* > 0.05), suggesting overall stability in diaphragmatic function across the transition.

When comparing patients according to extubation outcome (Table 3), several important distinctions emerged. Children with failed extubation had significantly lower BMI (13.2 ± 4.7 kg/m^2^) than those with successful extubation (17.4 ± 5.3 kg/m^2^; *p* = 0.039). The mean duration of intubation was also longer in the failure group (14.2 ± 13.6 days) compared with the success group (7.7 ± 5.6 days; *p* = 0.049). A smaller air-around-tube difference—defined as the gap between the laryngeal air-column diameter and the outer diameter of the endotracheal tube—was observed in failed cases (0.12 ± 0.09 cm) versus successful ones (0.22 ± 0.13 cm; *p* = 0.032), suggesting a tighter tube fit and potential risk of airway edema.

**Table 3:** Comparison between successful and failed extubation groups

Variable	Failed extubation (*n* = 12)	Successful extubation (*n* = 18)	*p*-value
Age (months)	54.3 ± 65.2	49.7 ± 55.7	0.835
Male sex *n* (%)	9 (75.0)	13 (72.2)	0.866
BMI (kg/m^2^)	13.2 ± 4.7	17.4 ± 5.3	0.039*
Air around the tube (cm)	0.12 ± 0.09	0.22 ± 0.13	0.032*
LUS aeration score	22.8 ± 5.8	17.0 ± 5.1	0.113
Diaphragmatic thickening fraction (%)	29.3 ± 11.3	52.3 ± 19.9	0.001**
Diaphragmatic excursion (cm)	0.48 ± 0.12	1.06 ± 0.23	< 0.001**
Time-to-peak inspiratory amplitude (s)	0.30 ± 0.13	0.33 ± 0.14	0.635
Days of intubation	14.2 ± 13.6	7.7 ± 5.6	0.049*
Respiratory support after extubation *n* (%)	HFNC 6 (50.0), CPAP 6 (50.0)	HFNC 8 (44.4), CPAP 3 (16.7), low flow 3 (16.7), none 4 (22.2)	0.061
Post-extubation stridor *n* (%)	8 (66.7)	8 (44.4)	0.227

*BMI* body mass index, using: Independent *T*-test for mean ± SD, *p*-value > 0.05 is insignificant; *p-value < 0.05 is significant; **p-value < 0.01 is highly significant

Respiratory muscle performance emerged as the strongest discriminator between groups. The mean diaphragmatic thickening fraction (DTF) was significantly lower in the failed group (29.3 ± 11.3%) compared with the success group (52.3 ± 19.9%, *p* = 0.001). Similarly, diaphragmatic excursion (DE) was markedly reduced among patients who failed extubation (0.48 ± 0.12 cm vs. 1.06 ± 0.23 cm, *p* < 0.001). The lung aeration score was numerically higher in the failed group (22.8 ± 5.8) than in the success group (17.0 ± 5.1), consistent with greater aeration loss, although this difference did not reach statistical significance (*p* = 0.113).

Multivariable regression analysis (Table 4) confirmed that higher BMI (*β* = 0.036, 95% CI: 0.004–0.068, *p* = 0.039); greater DTF (*β* = 0.014, 95% CI: 0.006–0.022, *p* = 0.001); larger DE (*β* = 0.970, 95% CI: 0.580–1.360, *p* < 0.001); and wider air-around-tube difference (*β* = 2.126, 95% CI: 0.372–3.880, *p* = 0.018) were independently associated with extubation success. The duration of intubation lost significance in the adjusted model, suggesting its effect was mediated by diaphragmatic dysfunction rather than duration itself.

**Table 4:** Multivariate regression analysis for predictors of extubation success

PPredictor	β (95% CI)	*p*-value
BMI	0.036	0.039*
Diaphragmatic thickening fraction	0.014	0.001**
Diaphragmatic excursion	0.970	< 0.001**
Air around the tube	2.126	0.018*
Days of intubation	− 0.016	0.079

*BMI* body mass index, *p*-value > 0.05 is insignificant; *p-value < 0.05 is significant

Receiver-operating-characteristic (ROC) analysis (Table 5) further demonstrated the diagnostic accuracy of the ultrasound parameters. Diaphragmatic excursion > 0.55 cm and thickening fraction > 29% yielded the highest predictive performance (AUC = 0.98 and 0.91, respectively; both *p* < 0.001). An LUS aeration score < 16.5 predicted extubation success with 80% sensitivity and specificity (AUC = 0.81, *p* = 0.038). A smaller laryngeal air-column width difference (LACWD < 0.17 cm) predicted extubation failure with 83.3% specificity (*p* = 0.025).

**Table 5:** ROC curve analysis for predictors of extubation failure

PPredictor	Cutoff	AUC	Sensitivity	Specificity	PPV	NPV	*p*-value
BMI	** < **14.79	0.727	100%	63.6%	50.1%	83.1%	0.043*
Air around the tube	** < **0.120	0.706	75.0%	66.7%	44.0%	93.0%	0.060
Air width difference	< 0.165	0.745	66.7%	83.3%	63.3%	85.4%	0.025*
Diaphragmatic thickening fraction	< 29.0	0.914	88.9%	66.7%	100%	91.3%	< 0.001**
Diaphragmatic excursion	< 0.55	0.979	100%	91.7%	83.8%	100%	< 0.001**
Time to peak inspiratory amplitude	< 0.215	0.546	88.9%	33.3%	36.4%	87.5%	0.672
Lung ultrasound score	> 16.5	0.810	80.0%	80.0%	63.2%	90.3%	0.038*
Days of intubation	> 5.5	0.687	83.3%	55.6%	44.6%	88.6%	0.086

*BMI* body mass index, *PPV* positive predictive value, *NPV* negative predictive value. *p*-value > 0.05 is insignificant; *p-value < 0.05 is significant

Overall, these findings indicate that diaphragmatic ultrasound—particularly measurements of excursion and thickening fraction—provides the most robust predictors of extubation outcome in mechanically ventilated children, while lung and laryngeal ultrasound offer complementary, airway- and aeration-specific insights.

## Discussion

Predicting extubation success in critically ill children remains one of the most challenging tasks in pediatric intensive care. Extubation readiness is primarily a clinical decision integrating hemodynamic stability, gas exchange, neurological recovery, and resolution of the primary disease. Point-of-care ultrasound (POCUS) should therefore be regarded as a complementary tool that augments rather than replaces bedside evaluation. In this study, ultrasound findings were interpreted alongside clinical parameters such as spontaneous breathing trial performance, cough strength, gas exchange, and secretion burden, reflecting real-world practice and aligning with current international recommendations for multimodal extubation assessment.

This pilot study evaluated a multi-organ ultrasonographic approach—combining diaphragmatic, lung, and laryngeal imaging—to enhance extubation readiness assessment. The diaphragm emerged as the key determinant of success: both diaphragmatic excursion (DE) and thickening fraction (DTF) were strong predictors, with DE > 0.55 cm and DTF > 29% yielding excellent discrimination (AUC 0.98 and 0.91). These findings are consistent with prior pediatric and adult studies showing that impaired diaphragmatic contractility is a principal cause of weaning failure [11–13]. Although absolute thresholds vary by age and ventilation strategy, our observed cut-offs fall within previously reported pediatric ranges [14, 15]. The limited predictive value of time-to-peak inspiratory amplitude (TPIA) may reflect anatomical and physiological differences in children and variability in spontaneous breathing trial protocols [16, 17].

The lung ultrasound (LUS) aeration score provided complementary information by identifying aeration loss associated with increased work of breathing and fluid overload. Scores > 16.5 were linked to extubation failure, in line with previous pediatric [14, 18, 19] and adult [20, 21] data. These findings reinforce the 2025 ESICM–ESPNIC recommendations advocating standardized 10- or 12-zone scoring to improve reproducibility and age-appropriate interpretation.

Laryngeal ultrasound also contributed meaningfully by detecting upper airway narrowing. A smaller laryngeal air-column width difference (LACWD) correlated with extubation failure, consistent with periglottic edema mechanisms and earlier work associating narrow air columns with post-extubation stridor or reintubation [22–24]. No significant association was found between tube size mismatch and laryngeal ultrasound parameters; however, incorrectly sized tubes were more frequently linked to post-extubation stridor severity.

It is important to note that at the time of data collection, the 12-zone LUS scoring protocol was applied to all patients, including infants. The updated 2025 ESICM–ESPNIC international guidelines 7 now recommend a 10-zone LUS aeration score for infants < 18 months to improve feasibility. Our cohort pre-dated this recommendation; recalculating using the 10-zone system would not materially alter relative differences between success and failure groups.

The relatively high extubation failure rate (40%) likely reflects our tertiary-referral case mix, which included a large proportion of chronically ill and complex patients. This underscores the importance of validating our findings across different clinical settings and acuity levels.

This multi-organ ultrasonographic approach provides a more complete physiological framework: LUS alone cannot distinguish parenchymal dysfunction from airway obstruction, whereas combining diaphragmatic, lung, and laryngeal imaging captures the principal contributors to post-extubation respiratory failure. Although hemodynamic factors were not evaluated, integrating cardiac ultrasound in future work could further strengthen predictive models.

A key limitation of the present study is the lack of etiological classification of extubation failure (airway obstruction vs parenchymal vs neuromuscular causes). The study’s goal was to evaluate overall predictive performance, but future research should prospectively categorize failure causes to refine organ-specific ultrasound predictors.

Lower BMI independently predicted failure, highlighting the interplay between nutritional reserve, respiratory muscle performance, and weaning capacity. Tube type (cuffed vs uncuffed) and age were not associated with outcomes, consistent with evidence supporting the safety of cuffed tubes in pediatric ventilation [25, 26]. Although longer intubation duration was associated with failure in univariate analysis, it lost significance after adjustment, suggesting mediation by diaphragmatic dysfunction—consistent with data on ventilator-induced diaphragmatic weakness [27].

## Limitations

This single-center pilot study with a small sample size limits generalizability and statistical power. The relatively high extubation failure rate likely reflects our tertiary-referral population of complex, chronically ill children. All ultrasound scans were performed by a single expert radiologist, ensuring consistency but precluding inter-observer validation; this reflects transitional practice, with the goal of implementing intensivist-led POCUS following structured training. Hemodynamic assessment was not included, and thus cardiovascular factors could not be analyzed. The uniform 12-zone LUS score was applied to all age groups before the 2025 ESICM–ESPNIC recommendation to use a 10-zone system for infants, though this is unlikely to alter the main findings. Finally, while the 24-h peri-extubation window was consistent with prior pediatric studies, a shorter interval may enhance predictive accuracy and should be standardized in future work.

## Clinical implications and recommendations

Despite its exploratory nature, this pilot study supports the feasibility of incorporating multi-organ ultrasound into routine extubation assessment in pediatric intensive care units. Diaphragmatic excursion and thickening fraction may serve as key quantitative indicators of extubation readiness, complemented by lung ultrasound for parenchymal evaluation and laryngeal imaging for airway risk stratification. Implementing standardized protocols and structured training for intensivists will enhance bedside reproducibility and independence from radiology services. Future multicentre studies integrating cardiac and hemodynamic indices are needed to establish comprehensive, evidence-based ultrasound-guided weaning algorithms.

## Conclusion

Diaphragmatic ultrasound—particularly excursion and thickening fraction—demonstrated the highest predictive accuracy for extubation success, while lung and laryngeal ultrasound provided complementary information on aeration and airway patency. Together, these modalities form a holistic, non-invasive framework for extubation readiness assessment. Validation in larger, multicentre populations is warranted to refine diagnostic cut-offs and standardize their integration into pediatric critical care practice.

## Data Availability

The datasets used and/or analyzed during the current study are available from the corresponding author on reasonable request.

## Abbreviations

ABG: Arterial blood gas
AUC: Area under the curve
BMI: Body mass index
CPAP: Continuous positive airway pressure
DE: Diaphragmatic excursion
DTF: Diaphragmatic thickening fraction
ETT: Endotracheal tube
FMASU REC: Faculty of Medicine, Ain Shams University Research Ethics Committee
FWA: Federal Wide Assurance
HCO₃: Bicarbonate
HFNC: High-flow nasal cannula
LACWD: Laryngeal air column width difference
LUS: Lung ultrasound score
MV: Mechanical ventilation
NPV: Negative predictive value
PICU: Pediatric Intensive Care Unit
POCUS: Point-of-care ultrasound
PPV: Positive predictive value
ROC: Receiver operating characteristic
SBT: Spontaneous breathing trial
SD: Standard deviation
Te: Diaphragm thickness at end-expiration
Ti: Diaphragm thickness at end-inspiration
TPIA: Time to peak inspiratory amplitude
US: Ultrasound
